# Exo-nanomaterials in cancer immunotherapy: reprogramming the tumor immune microenvironment

**DOI:** 10.3389/fimmu.2025.1764525

**Published:** 2026-01-19

**Authors:** WeiJian Fang, EnDuo Qiu, Rui Liu, ShanLin Wang, TianFu Wang, YuMing Wang

**Affiliations:** Second Ward of Bone and Soft Tissue Tumor Surgery, Cancer Hospital of Dalian University of Technology, Cancer Hospital of China Medical University, Liaoning Cancer Hospital & Institute, Shenyang, Liaoning, China

**Keywords:** exosomes, extracellular vesicles, nanomaterials, solid tumors, tumor immunity

## Abstract

Immunotherapies such as checkpoint blockade, adoptive cell transfer and vaccines can induce durable responses, yet most solid tumors remain refractory because the tumor immune microenvironment (TIME) is both immunosuppressive and physically difficult to access. In parallel, extracellular vesicles (EVs) and synthetic nanomaterials have emerged as complementary immune messengers and programmable carriers. Exo-nanomaterials, hybrids that fuse EV membranes with synthetic cores, aim to unite EV biocompatibility and trafficking with the loading capacity, modularity and stimulus-responsiveness of engineered nanomaterials. Here, we summarize how exosomes shape the TIME by distributing checkpoint ligands, reprogramming myeloid cells and modulating antigen presentation, and how nanomaterials are engineered to improve tumor-localized delivery of innate agonists and vaccine cargos. We then outline major construction routes (coating, loading and mimetic fabrication) and design modules that enable cold-to-hot conversion, sensitization to checkpoint blockade, and delivery of neoantigen and nucleic-acid vaccines. Finally, we discuss key translational challenges, including standardization, mechanism deconvolution, scalable manufacturing and safety, and propose immune-by-design principles to guide reproducible, mechanism-grounded development toward durable immunotherapy in solid tumors.

## 1 Introduction

Immune checkpoint blockade, adoptive cell transfer and cancer vaccines have delivered durable benefit in a subset of patients, yet most solid tumors remain refractory because the TIME is heterogeneous, suppressive and physically hard to access. In practice, two constraints repeatedly co-exist: (i) immune dysfunction and adaptive resistance within the TIME, and (ii) insufficient delivery of immunomodulators into excluded tumor niches. Recent clinical and preclinical advances indicate that responses deepen when antigen supply, T-cell reinvigoration and cytokine support can be coordinated in space and time rather than delivered as diffuse systemic exposure (1, 2). Spatially segregated “hot” and “cold” regions can also co-evolve under treatment pressure, creating new bottlenecks even after an initial response (3, 4). These realities motivate carrier technologies, increasingly including hybrid exo-nanomaterials, that can localize, sequence, and sustain immune cues within excluded niches with spatiotemporal control.

Programmable nanomaterials offer engineering control over size, surface chemistry and release kinetics, enabling delivery of innate agonists, co-delivery of adjuvants with checkpoint modulation and modular vaccination strategies designed to convert “cold” tumors into inflamed lesions and mitigate antigen escape (5–7). However, many nanosystems still face biological-interface limitations: opsonization and reticuloendothelial sequestration, incomplete penetration into stromal-excluded regions and off-target innate activation, so physicochemical precision does not always translate into *in vivo* immunological specificity or acceptable safety.

EVs, including exosomes, represent a biologically native communication and transport layer with favorable biocompatibility and tissue navigation. EV-assisted vaccination and innate-adaptive crosstalk can enhance antitumor immunity, and engineered exosomes can locally modulate checkpoints to improve CD8^+^ T-cell function (8–10). Importantly, EV therapeutics are entering the clinic: engineered exosomes delivering KRAS^G12D-targeting siRNA demonstrated feasible tumor delivery with immunological correlates in a first-in-human phase-I study (11), and cross-kingdom vesicles further expand sourcing strategies and cargo space (12). Yet EV platforms remain constrained by limited payload capacity, batch heterogeneity, standardization challenges and scalable GMP manufacturing, together with unresolved long-term safety questions across diverse sources.

These complementary strengths and limitations are now converging into exo-nanomaterials, hybrids that fuse EV membranes with synthetic cores to combine biological navigation/targeting with high loading capacity and stimulus-controlled release. Examples such as inhalable EV delivery of IL-12 mRNA and membrane-coated nanoparticles bearing multiple immunostimulatory ligands illustrate how potent immune activation can be localized while limiting systemic toxicity (13, 14). We therefore posit that immune-by-design exo-nanomaterials represent a next-generation platform to overcome the dual challenges of TIME heterogeneity and delivery barriers for precise TIME remodeling in solid tumors.

Accordingly, this review maps key TIME barriers to actionable design requirements (Section 2), summarizes nanomaterial-enabled immunomodulatory strategies (Section 3), details exo-nanomaterial construction routes and design modules (Section 4), surveys major immunotherapy applications by functional outcomes (Section 5), and discusses translational bottlenecks including standardization, mechanism deconvolution, scalable manufacturing and safety (Section 6).

## 2 Tumor immunity and TIME barriers as design requirements for EV-enabled engineering

Solid tumors respond incompletely to ICIs, adoptive cell therapies and vaccines because antitumor immunity is constrained by a small set of actionable, spatially organized barriers in the TIME. Here we distill these bottlenecks into design requirements that later sections translate into nanomaterial, EV and exo-nanomaterial engineering choices. Rather than exhaustively cataloging cell types, we focus on barriers that most directly dictate whether immune cues can reach tumor nests, reprogram suppressive circuits and sustain effector function *in vivo*.

### 2.1 TIME barriers that most strongly constrain durable immunotherapy

#### 2.1.1 Barrier 1: suppressive myeloid-regulatory circuits and checkpoint reinforcement

A common TIME state is a suppressive, myeloid- and Treg-supported ecosystem that can maintain T-cell dysfunction even when effector cells are present. Checkpoint-linked regulatory programs can intensify this suppression, including PD-1-programmed Treg states (15), while macrophage-intrinsic PD-1 signaling can reinforce suppressive myeloid behavior through JAK2-STAT3 rewiring (16). Clinically relevant SPP1^+^ TAM programs align with CD8^+^ dysfunction and immunotherapy resistance (17), and suppressive myeloid burden can be causally tied to ICI failure; reducing MDSCs can directly rescue antitumor immunity and synergize with anti-PD-1 (18). Design implication: carriers should enable cell-selective and local delivery (e.g., TAM/MDSC/Treg targeting) to reprogram these circuits and reduce rebound suppression rather than relying on systemic exposure alone.

#### 2.1.2 Barrier 2: stromal-metabolic exclusion and hostile niches

Immune dysfunction is stabilized by tissue architecture and microregional conditions. CAF-driven ECM remodeling restricts CD8^+^ infiltration and can confer ICI resistance (19). Even where infiltration occurs, nutrient-poor and lactate-rich niches promote metabolic adaptations that erode durable effector function (20). Design implication: delivery systems should prioritize penetration, retention, and *in situ* activation in excluded or metabolically hostile regions (via size/charge tuning, matrix-aware strategies, and/or locoregional administration) to ensure immune cues actually operate where suppression is maintained.

#### 2.1.3 Barrier 3: impaired priming-presentation-effector coupling and dynamic resistance under treatment pressure

A second decisive bottleneck is the priming-presentation-effector loop, which can be throttled at multiple steps and can shift during therapy. cDC1 availability can gate ICI efficacy (21), and tumor-intrinsic restoration of antigen visibility (e.g., strengthening MHC-I machinery) can sensitize solid tumors to T cell-dependent immunotherapy (22); in patients, therapy-associated increases in B2M/HLA-A can track with response, reinforcing that antigen presentation is dynamic rather than fixed (23). Yet even with priming, persistent antigen exposure in suppressive niches drives exhaustion-like transcriptional programs (24), and resistance can emerge as distinct, selectable tumor-TIME programs under treatment pressure (25). Spatial organization is a unifying constraint across all three barriers, because it ultimately determines whether delivered cues can reach and operate within tumor nests; consistent with this, tumor–stroma interface modeling shows that stromal borders can slow immune motility and block deep infiltration (26). Design implication: platforms should be programmable and re-dosable, with spatially informed delivery/activation, to reinforce priming and antigen visibility while limiting exhaustion and adaptive resistance.

### 2.2 Exosomes: a mechanistic communication layer that can be exploited or reprogrammed

Exosomes (30–150 nm EVs) constitute a mobile communication layer that links tumor, stromal and immune niches. Importantly, immune escape can be encoded at the EV-sorting step: Munc13–4 promotes preferential PD-L1 loading into exosomes, and disrupting this pathway restores CD8^+^ function and improves checkpoint responsiveness *in vivo* (27). EVs can also impose exhaustion programs without direct tumor–T-cell contact; breast cancer EVs promote CD8^+^ exhaustion via TGF-β type II receptor signaling (28).

Beyond direct immune targeting, tumor exosomal tsRNA can induce fibroblast senescence and Galectin-9 secretion, amplifying tolerance through stromal reprogramming (29). Design implication: EV biology provides both (i) targets to neutralize EV-mediated suppression (e.g., EV PD-L1/decoy pathways) and (ii) templates for niche-local delivery when fused with programmable cores.

### 2.3 Tumor- versus immune-derived exosomes define both resistance modes and engineering blueprints

Tumor-derived exosomes (TEX) do not merely reflect suppression; they can actively amplify it and create resistance modes that classic dosing does not fully address. Exosomal PD-L1 can reinforce a Treg-M2 macrophage positive feedback loop (30), and TEX can blunt ICIs through antibody decoying that reduces functional drug availability at immune synapses (31). TEX also program innate dominance: exosomal miR-205 can drive M2 polarization via PTEN/PI3K–AKT–mTOR in ovarian cancer (32), and additional miRNA transfer routes sustain macrophage re-education in other tumor contexts (33). In parallel, TEX can expand suppressive pools by recruiting MDSCs via CXCR4 upregulation through TLR2/NF-κB signaling (34), remodel immune entry rules by reprogramming lymphatic endothelial cells toward IDO-linked tolerance (35), and even delete effector function directly; patient plasma small EVs can induce intrinsic apoptosis in activated T cells (36). These observations together motivate hybrid exo-nanomaterial solutions that combine EV-like trafficking and biocompatibility with programmable payload control, aiming to neutralize EV-mediated resistance (e.g., decoys), reprogram suppressive myeloid and stromal compartments, and protect effector function.

Conversely, immune-cell–derived EVs and EV vaccination strategies provide constructive “design blueprints” for therapeutic engineering. Circulating NK-cell exosomes can exhibit antitumoral activity in patient-derived settings (37), and cytokine conditioning can enhance NK-exosome cytotoxic loading and killing potency (38), implying that EV function can be tuned via controllable input rules. DC-derived EVs can be engineered to co-present IL-12 and an anti-CTLA-4 format on their surface to strengthen CD8^+^ activation (39), while CAR-T-derived EVs can retain antigen specificity and contribute directly to antineoplastic effects (40) with reports of potent activity and lower systemic toxicity than whole-cell approaches (41). Finally, *in vivo* EV vaccination that routes antigens into EV production pathways can elicit strong tumor-specific CD8^+^ immunity and near-complete tumor control in stringent dual-antigen models, underscoring how EV biology can be repurposed as a priming factory to address antigen presentation bottlenecks (42).

### 2.4 Design principles for exo-nanomaterials toward TIME remodeling: a mapping framework

Rather than cataloging exo-nanomaterials by material class, we use an “immune-by-design” mapping that connects TIME barriers to engineering levers, intended immunological effects, and minimal *in vivo* readouts. This rubric links physicochemical and pharmaceutic parameters (size/charge/shape, membrane source, surface ligands, stimulus-responsiveness, co-delivery and release kinetics, and administration route) to concrete bottlenecks (spatial exclusion, suppressive myeloid circuits, impaired antigen presentation, and systemic toxicity) and specifies what constitutes adequate *in vivo* evidence for TIME remodeling. **Table 1** is used as the organizing scaffold and evaluation yardstick throughout Sections 3–5, enabling side-by-side comparison of systems by barrier targeted, design logic, mechanistic readouts, and translational trade-offs, rather than by materials taxonomy.

**Table 1:** Mapping TIME barriers to design levers for exo-nanomaterials and expected immunological outcomes.

TIME barrier	Key design levers	Remodeling goal	Minimal readouts
Immune exclusion/dense ECM	50–150 nm; near-neutral; local retention	deeper penetration; ↑CD8^+^ entry	penetration/retention; CD8^+^ depth; CAF/ECM
Hypoxia/acidity	pH-triggered release	protect effectors; weaken myeloid suppression	hypoxia/pH; TAM state; cytokines
TAM/MDSC dominance	myeloid targeting; “activate + de-suppress”	M2→M1; ↓MDSC; ↑APC function	TAM/MDSC; MHC/IL-12; CD8^+^ function
Weak priming/cross-presentation	antigen+adjuvant co-delivery; LN/DC targeting	↑DC maturation; ↑antigen-specific CD8^+^	LN uptake; DC markers; tetramer^+^ CD8^+^
STING delivery limits	EV-assisted cytosolic access; localized kinetics	sustained type-I IFN program	IFN-β/CXCL10; APC activation; safety
Exosomal PD-L1/decoy resistance	PD-L1 knockdown; block EV PD-L1	restore CD8^+^ activity; sensitize ICB	EV PD-L1; exhaustion; ICB response
“Cold” tumor (low danger)	PTT/PDT/SDT; ROS/ferroptosis modules	ICD → DC activation → infiltration	CRT/HMGB1/ATP; DC maturation; CD8^+^
Systemic toxicity	local delivery; gated release	widen therapeutic window	serum cytokines; organ tox; weight
Scale-up/heterogeneity	mimetics; microfluidics; QC	reproducibility; GMP readiness	size/ζ; EV markers; batch CV

## 3 Nanomaterials for immunomodulation

**Figure 1:**
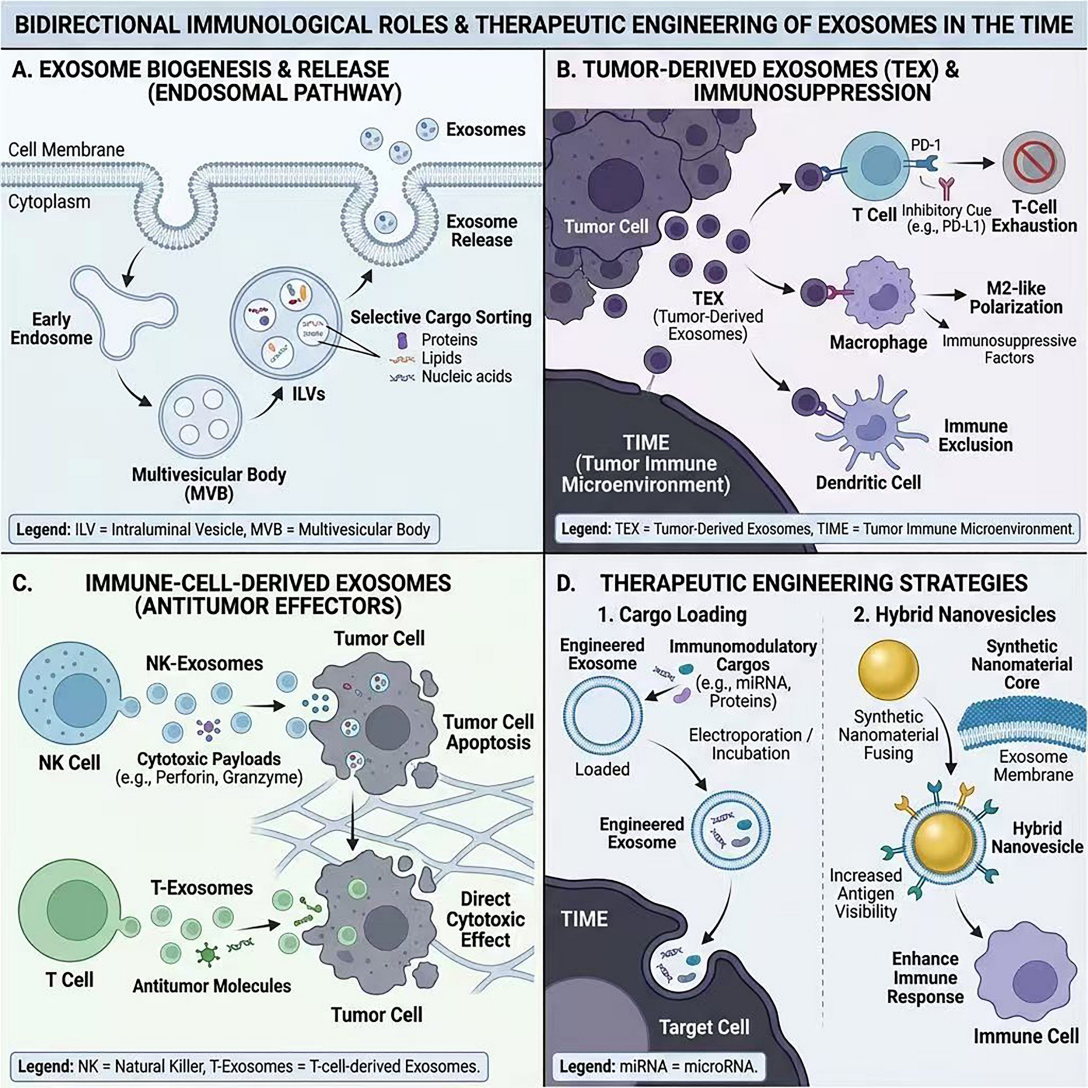
Bidirectional immunological roles and therapeutic engineering of exosomes in the TIME. **(A)** Exosome biogenesis and release via the endosomal pathway: MVBs form ILVs through selective cargo sorting and release 30–150 nm exosomes. **(B)** Tumor-derived exosomes (TEX) disseminate inhibitory cues (e.g., PD-L1), skew macrophages toward M2-like states, and promote T-cell exhaustion, DC dysfunction, and immune exclusion. **(C)** Immune-cell-derived exosomes can mediate antitumor effects: NK exosomes deliver cytotoxic proteins, T-cell exosomes convey antigen-specific effector signals, and DC exosomes enhance cross-presentation and T-cell priming; CAR-T exosomes may retain antigen specificity with lower CRS risk. **(D)** Therapeutic engineering of exosomes: cargos (siRNA/mRNA, cytokines, STING agonists) and surface functionalization enable tumor homing. Fusion with synthetic cores yields hybrids that combine biocompatibility with programmable delivery to enhance antigen visibility, innate sensing, myeloid repolarization, and effector responses.

Nanomaterials have become central to tumor immunology because they can be engineered to (i) deliver immune drugs precisely into the TIME, (ii) trigger innate sensing pathways, and (iii) rewire suppressive cellular circuits that keep tumors “cold.” Unlike small-molecule or antibody drugs that rely on passive diffusion and broad systemic exposure, nanosystems are tunable in size, shape, surface chemistry and release behavior, so that where immune activation happens and how long it lasts can be programmed rather than left to chance. This design freedom is now being used to address the “last mile” of immunotherapy—getting the right signal into the right TIME niche at the right time. This nanomaterial logic also motivates exosome-guided or exosome-hybrid delivery concepts discussed earlier, where natural vesicle trafficking and engineered payloads can be combined to modulate the TIME (**Figure 1**).

### 3.1 Nanodelivery improves the “on-target rate” of immunotherapeutics

A core limitation of many immunotherapies is that only a small fraction of the injected agent reaches tumors or the correct immune cell compartments. Nanomaterials increase this “on-target rate” by stabilizing fragile cargos, prolonging circulation and enabling both lymphoid trafficking and tumor deposition. Antigen-capturing nanoparticles (AC-NPs) that scavenge therapy-released tumor antigens and ferry them into cross-presenting DCs, related biomimetic nanovaccines cloaking immunogenic polymer cores with tumor membranes to broaden the antigen repertoire and preserve native epitope conformation, and intratumoral STING-activating nanovaccines built on synthetic polymer adjuvants that more effectively reprogram draining lymph nodes and the local TIME than systemic dosing collectively exemplify how nanodelivery can script the location and quality of immune activation rather than simply raise drug concentration (43–45).

### 3.2 Immune-activating nanomaterials: delivering and amplifying innate agonists

A second thrust is to design nanomaterials that directly engage innate immune pathways such as STING, TLR7/8 and cGAS-related sensors or that deliver these agonists into the cytosol with controlled kinetics. Polyvalent PC7A-based polymeric nanoparticles show how material properties can encode signal duration: by undergoing immune-dependent phase transitions they prolong STING activation and type-I IFN programs, with polymer architecture rather than dose alone dictating the temporal profile (46). Nanoparticles that encapsulate cyclic dinucleotide agonists in polymersome shells build on this logic, markedly improving pharmacokinetics, shifting biodistribution toward tumors and thereby opening an intravenous therapeutic window for systemic STING immunotherapy and checkpoint combinations (47). Chemically programmed STING-activating nano-liposomes (SAProsomes), in which agonists are covalently embedded in the liposomal membrane, further coordinate release and reduce systemic inflammation while maintaining strong intratumoral interferon responses (48).

Innate agonists can also be positioned in space using physical targeting and hybrid materials. Nucleotide nanocomplexes displayed on ultrasound microbubbles enable image-guided vascular extravasation and local cytosolic delivery of cGAMP, sharply enhancing STING activation in otherwise poorly penetrant tumors (49). Dual-STING-activating nanosystems such as D-SAM integrate direct receptor agonism with amplified intracellular trafficking, broadening the fraction of tumors that respond and helping to overcome resistance to checkpoint blockade in preclinical models (50). Hybrid manganese nanoparticles, which both debulk cancer stem cell populations and release Mn²^+^ as a cofactor for cGAS–STING signaling, couple physical tumor reduction to innate “ignition” in highly refractory carcinomas (51). Finally, implantable sandwich-structured dual-drug depots that release a non-nucleotide STING agonist first and an apoptosis inducer second create a staged local cascade particularly suited to TIME re-education after debulking surgery and to the prevention of post-surgical recurrence (52). Across these designs, nanomaterials converge on a shared logic: they enhance cytosolic access for agonists, shape the kinetics of innate signaling and localize activation to TIME niches where antigen presentation and T-cell priming are most effective.

### 3.3 Nanomaterials remodel suppressive TIME circuits

Even with strong innate triggers, immunotherapy fails if suppressive myeloid and stromal programs remain intact, so nanomaterials are increasingly being used to edit TIME cell states directly. Albumin-based nanoparticles co-delivering the photothermal sensitizer IR820 and the SHP2 inhibitor SHP099 can reorient M2-like TAMs toward M1-like phenotypes, restore macrophage phagocytosis and reinforce cytotoxic T-cell recruitment, jointly shifting the TIME from suppressive to inflamed while limiting systemic exposure to the inhibitor (53). In hepatocellular carcinoma, lipid-coated tannic-acid nanoparticles carrying a CXCR4 antagonist (807-NPs) disrupt the hypoxia-driven CXCL12/CXCR4 axis that supports M2 polarization and T-cell exclusion, yielding marked TAM repolarization and enhanced sensitivity to PD-1 blockade (54). Polymeric micellar nanoparticles that co-formulate a TLR7/8 agonist with a PI3Kδ inhibitor extend this concept into the context of radiotherapy: by reducing MDSCs and tissue-resident Tregs while promoting type-1 macrophages and adaptive T- and B-cell expansion, they “lock” immunostimulatory and anti-suppressive cues into a single spatiotemporal package and help prevent the TIME from re-establishing resistance after irradiation (55). In each case, nanomaterials are not simply adding more stimulatory signals but actively subtracting suppressive ones within the same anatomical compartment.

### 3.4 Material class–immune effect relationships

Finally, different nanomaterial classes tend to produce distinct immune signatures because they differ in uptake pathways and degradation kinetics. PPS-based polymeric nanoparticles carrying TLR7/8 agonists can be tuned to selectively prolong DC activation, maintaining IL-12-dominated priming while limiting systemic cytokine spillover; adjusting their degradation rate allows innate activation windows to be aligned with antigen presentation and T-cell priming (56). By contrast, amphiphilic prodrug self-assemblies of the TLR7/8 agonist R848 form carrier-free nanostructures whose disassembly is tied to intracellular enzymatic or redox environments, creating high local receptor potency with reduced off-target stimulation and minimal excipient burden (57). In practical terms, polymeric carriers excel at programmable multi-cargo release, whereas prodrug or coordination assemblies boost per-particle immunogenicity and simplify formulations. Recognizing these class-specific tendencies provides a useful framework for rational exo-nanomaterial design in subsequent sections.

## 4 Engineering exo-nanomaterials

### 4.1 Fusion rationale and complementary advantages

Exo-nanomaterials are hybrids that integrate natural exosomes (or exosome-like EVs) with synthetic nanomaterials into a single therapeutic unit. The logic is pragmatic: exosomes are biologically “native” carriers with low immunogenicity, long circulation, and inherent roles in immune communication, but their translation is often limited by low yield, heterogeneity, and modest cargo capacity. Scalable top-down production of exosome-mimetic nanovesicles by membrane extrusion can boost vesicle yield while preserving much of the parent-cell surface identity, helping address the manufacturing bottleneck (58). Reproducible wrapping of membranes onto cores has also been enabled by microfluidic sonication–driven fusion (59).

**Figure 2:**
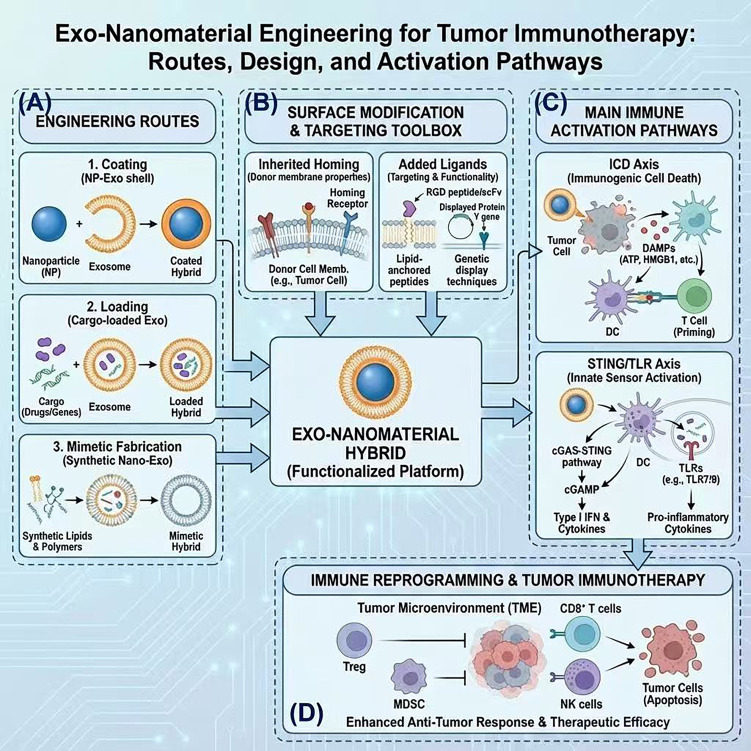
Exo-nanomaterial engineering for tumor immunotherapy: routes, surface design and immune activation pathways. **(A)** Engineering routes for constructing exo-nanomaterial hybrids. Coating strategies wrap pre-formed nanoparticle cores with exosomal membranes to generate NP–Exo core–shell hybrids; loading approaches introduce drugs or nucleic acids into exosomes to obtain cargo-loaded vesicles; mimetic fabrication uses synthetic lipids and polymers to build nano-exosome analogs with exosome-like size, composition, and functionality. **(B)** Surface modification and targeting toolbox. Inherited homing is conferred by donor-cell membranes (e.g., tumor-cell membranes) that carry integrins and other homing receptors. Additional ligands are introduced via lipid-anchored peptides, RGD peptides, Fc fragments, or genetically displayed targeting proteins, enabling refined tumor tropism and functionalization. **(C)** Main immune-activation pathways engaged by exo-nanomaterial hybrids. Immunogenic cell death (ICD) of tumor cells releases damage-associated molecular patterns (DAMPs; such as ATP and HMGB1), promotes DC activation, and primes tumor-specific T cells. In parallel, STING and TLR pathways are activated through cGAS–STING signaling and TLR engagement, inducing type I interferons and pro-inflammatory cytokines. **(D)** Immune reprogramming and tumor-immunotherapy outcomes. By integrating these routes and pathways, exo-nanomaterial hybrids remodel the TIME, decrease Tregs and MDSCs, enhance the activity of CD8^+^ T cells and NK cells, and promote tumor cell apoptosis, ultimately leading to strengthened antitumor immune responses and improved therapeutic efficacy.

An overview of exo-nanomaterial engineering routes, surface design strategies and immune activation pathways is illustrated in **Figure 2**.

On the other side, synthetic nanomaterials bring what exosomes usually lack: high loading density, precise control over size/shape/charge, and stimulus-responsive behaviors. When fused, the exosome shell supplies “self” markers and targeting ligands, while the core supplies programmable physics. Direct membrane-fusion techniques—like microfluidic sonication—allow exosome membranes to be wrapped onto diverse nanoparticle cores without fully disrupting the vesicle architecture, which is essential for reproducible hybrid assembly.

Functionally, the hybrid often outperforms either component. For instance, brain-targeted exosome-mimetic cell membrane nanovesicles use an internal nanocore to carry therapeutic oligonucleotides, while the exosome-mimetic cloak improves blood–brain barrier penetration and glioblastoma homing, resulting in higher intratumoral drug accumulation and better survival than free oligonucleotides; similarly, integrate chemotherapeutic and photothermal agents within a vesicular shell, coupling strong photothermal/chemo capacity with immune camouflage to amplify tumor penetration and tumor cell killing (60, 61). Overall, fusion is not a cosmetic add-on; it is a strategy to co-opt biology for navigation and physics for controllable killing/immune priming.

### 4.2 Three construction routes: coating, loading, and mimetic fabrication

#### 4.2.1 Route I — coating (exosome membrane cloaking)

In coating strategies, a pre-fabricated nanoparticle (organic, inorganic, or hybrid) is wrapped with intact exosomes or purified exosomal membranes to form a core–shell structure. This route is ideal when the core contributes a strong modality (photothermal, magnetic, catalytic, radiosensitizing), while the shell contributes stealth and tropism. Exosome-coated Prussian Blue nanoparticles for glioblastoma exemplify this design, with a Prussian Blue core providing photothermal conversion and a tumor-derived exosome shell conferring glioma targeting and immune evasion–bypassing properties (62). Another coating-based strategy is an *in situ* sprayed nanovaccine that disorganizes the Golgi apparatus and suppresses exosomal PD-L1 in the postsurgical tumor bed, thereby enhancing local antigen presentation and improving postoperative melanoma immunotherapy (63).

#### 4.2.2 Route II — loading (intraluminal or interfacial cargo insertion)

Loading routes start from intact exosomes and introduce therapeutic agents into the lumen or bilayer. Methods include electroporation, freeze–thaw, surfactant permeabilization, or parental-cell pre-loading (cells “pack” cargo into exosomes before secretion). This route is especially strong for soluble immune modulators that need protection and tumor retention. ExoSTING, an extracellular vesicle formulation loaded with a cyclic-dinucleotide STING agonist, efficiently traffics to antigen-presenting cells and drives strong type-I IFN responses compared with free agonist (64). Exosome–liposome hybrids co-delivering triptolide and miR-497 further demonstrate how an exosomal bilayer can act as a biological “outer layer” that stabilizes and retargets a high-capacity lipid core, overcoming chemoresistant ovarian cancer with improved safety (65). In a vaccine context, extracellular-vesicle–hybrid plasmid-loaded lipid nanovesicles (Lipo@HEV) package plasmid antigens and adjuvants into a vesicular nanovaccine that drains to lymph nodes and elicits synergistic cancer immunotherapy (66).

#### 4.2.3 Route III — mimetic fabrication (top-down/bottom-up exosome analogs)

Mimetic routes reduce reliance on naturally secreted exosomes as the final carrier. Instead, they produce exosome-like shells by membrane extrusion or by assembling synthetic vesicles and inserting selected surface proteins or lipids. The main advantages are scalability and tighter compositional control. For example, exosome-mimetic nanovesicles generated by serial extrusion can be decorated with superparamagnetic iron oxide nanoparticles (SPIONs) and loaded with TNF-α, enabling magnetic guidance to cancer cell membranes where localized cytokine release induces potent cell death while limiting systemic toxicity (67). Mimetic logic is also expanding into route-specific delivery, such as inhalable nanovesicles loaded with a STING agonist that home to lung tumors, activate STING within the pulmonary microenvironment, and significantly enhance CAR-T-cell activity against solid tumors in the lung (68). Together, these three routes are not mutually exclusive—many advanced systems mix them (for example, mimetic shells combined with intraluminal loading).

#### 4.2.4 Critical comparison and translational outlook across construction routes

While coating, loading, and biomimetic fabrication are often introduced as parallel options, they differ substantially in immune potency, safety liabilities, and CMC/GMP readiness. Coating (membrane cloaking of a well-defined synthetic core) is typically the most modular and formulation-stable route, because core composition, size distribution, and release behavior can be tightly controlled, and membrane wrapping mainly tunes biological interfacing and tropism (69). Its main translational risks are membrane-source safety and heterogeneity (donor cell identity, adventitious agents, residual bioactive proteins) and the need to define CQAs that capture membrane integrity and functional ligand display, consistent with community standardization guidance for EV characterization and reporting (70). In addition, incomplete or non-uniform membrane coverage can shift uptake and *in vivo* performance, making coating completeness and membrane integrity practical release attributes that require fit-for-purpose assays and process control (71).

Loading (intraluminal/interfacial cargo insertion into intact EVs) can achieve high biological relevance and strong immune activity for nucleic acids or cytokines, but it is often the most variable route, as loading efficiency, membrane damage/aggregation, cargo leakage, and batch heterogeneity complicate dose definition and reproducibility, motivating stricter method transparency and multi-parameter characterization aligned with updated EV best practices (72). Biomimetic fabrication improves scalability and compositional control by reducing reliance on naturally secreted EVs, enabling tighter specification of lipid/protein content and more straightforward QC release criteria; however, it may trade off some native trafficking features and can introduce new immunogenicity or clearance profiles if “exosome-like” surfaces are incompletely recapitulated (73). Overall, coating and biomimetic routes with standardized membrane sources and clearly defined synthetic cores are generally closer to GMP translation, whereas loading-forward designs may deliver higher immune potency but demand more stringent process controls and orthogonal QC to ensure consistency and safety.

### 4.3 Drug-loading and functionalization modules

After selecting a construction route, the choice of payload determines how a hybrid system reshapes the TIME. At the nucleic acid level, a pivotal direction is to use engineered exosomes to deliver RNA interference or gene-editing cargos. Preclinical work has already shown that exosomes engineered to deliver siRNA against mutant KRAS can achieve on-target pathway inhibition and tumor control in pancreatic cancer models, setting the conceptual stage for clinical translation (74). Building on this logic, many platforms now embed small, labile immune agonists—such as STING, TLR and cGAS–STING-axis activators—into exosomal or exosome-mimetic vesicles so that these molecules are protected during circulation, released where needed, and translated into heightened antigen presentation, elevated CXCL10 and IFN-β, and more robust CD8^+^ T-cell priming.

A complementary strategy is to exploit the membrane itself as an “active shell.” Exosomes derived from immune cells can carry intrinsic antitumor instructions through their surface receptors and cargo. Photosensitive hybrid γδ-T-cell exosomes, for example, insert a photosensitizer into γδ-T-derived vesicles so that light-triggered ROS induces immunogenic cell death, while γδ-T exosomal proteins simultaneously stimulate NK and CD8^+^ T-cell activity, producing both strong local tumor control and enhanced systemic antitumor immunity (75). Beneath these biological interfaces, metallic or oxide nano-cores can be introduced to add catalytic ROS generation, ferroptosis enhancement or photothermal conversion. When such cores are wrapped in an exosomal shell, they concentrate within tumors and “turn immune death on” without the usual off-target toxicity of free nano-agents; in this view, the inorganic core functions as a compact physical engine, whereas the exosomal surface negotiates biological access and immune dialogue.

### 4.4 Targeting and stimulus-responsive release engineering

Targeting in exo-nanomaterials works at two coupled levels. First, hybrids inherit homing from donor membranes (integrins, tetraspanins, chemokine receptors). Second, they add ligands through lipid-anchoring, click chemistry, or genetic display in parental cells. A chimeric exosome platform functionalized with STING activation for personalized glioblastoma immunotherapy shows that targeting and immune activation can be optimized together: the engineered exosomal membrane drives glioma homing, while the internal STING-activating cargo induces strong type-I IFN signaling and deep TIME reprogramming (76).

Stimulus-responsive release then decides when and where payloads unlock. Light-triggered systems are particularly mature: designer exosomes that co-deliver chemotherapeutics, gene cargos and photothermal agents can use near-infrared irradiation to induce local hyperthermia and controlled drug release, achieving efficient tumor-targeted chemo/gene/photothermal combination therapy (77). Endogenous triggers are equally important for deep lesions. Acidity-activatable dynamic hybrid nanoplatforms derived from M1 macrophage EVs remain stable in circulation but reconfigure in the acidic tumor microenvironment, boosting triple-synergistic cancer immunotherapy through checkpoint enhancement, myeloid reprogramming and effector T-cell activation (78).

Conceptually, the best hybrids orchestrate a tightly coupled cascade in which an initial stimulus leads to tumor cell killing, the release of tumor antigens, the activation of innate immune pathways and, ultimately, the expansion of adaptive antitumor responses. It is this closed immunological loop that transforms a nanomedicine into a genuine nano-immunotherapy.

## 5 Immunotherapy applications of exo-nanomaterials

Exo-nanomaterial platforms described above have now been tested across multiple tumor types and immunotherapy settings. To connect engineering logic with functional outcomes, **Table 2** groups representative designs by immunological application rather than by individual construct.

**Table 2:** Representative exo-nanomaterial platforms for cancer immunotherapy.

Application	Exo-nanomaterial design & conceptual summary	Main tumor models	Representative references
Turning “cold” tumors “hot”	Photo-activated and ferroptosis-augmented exosome(-coated) hybrids (including BBB-crossing nanomicelles and exosome-cloaked chemo/photothermal nanoparticles) that induce ICD, increase DAMP release, promote DC maturation and facilitate CD8^+^ T-cell infiltration in previously non-inflamed lesions	Breast cancer, glioblastoma and other solid tumors	(74–78)
Sensitizing ICI and reversing resistance	Exosome or exosome-like vesicles delivering PD-L1 siRNA or ferroptosis inducers to curb PD-L1–rich tumor exosomes, reprogram TAMs/MDSCs and create a less suppressive, more checkpoint-responsive TIME	Multiple transplantable and spontaneous solid-tumor models	(79, 80)
Tumor vaccines and personalized antigen delivery	DC-derived exosome vaccines loaded with neoantigenic peptide nanocomplexes that enhance lymph-node targeting, improve cross-presentation and drive robust neoantigen-specific CD8^+^ T-cell expansion for personalized vaccination	Dual-neoantigen breast-like tumor models and related solid tumors	(81)
Combination therapies (photo/SDT/ferroptosis + immunity)	Thermosensitive exosome–liposome hybrids and photo/sonodynamic exo-hybrids that co-deliver chemotherapeutics and immunomodulators, coupling local cytotoxicity (photothermal/PDT/SDT/ferroptosis) with *in situ* immune stimulation in deep or metastatic lesions	Metastatic peritoneal carcinoma and other solid tumors	(77, 82)
Evidence across tumor types and organ-specific TIMEs	Neural stem-cell exosomes carrying CpG-STAT3 antisense oligonucleotides, exosome-like photo-immunotherapy nanosystems in HCC and tumor-exosome-bionic Type-I AIE nanozymes illustrating ICD-driven photo-immunotherapy and TIME remodeling across diverse indications	Glioblastoma, hepatocellular carcinoma, melanoma, breast cancer, colon cancer and others	(83–85)

### 5.1 Turning “cold” tumors “hot”

A core promise of exo-nanomaterials is that they can start immunity where tumors are silent. Cold tumors typically lack strong danger signals, so DCs stay immature and T cells fail to enter or persist. Exosome-hybrid platforms address this at two levels: (i) immunogenic tumor killing that releases antigens and DAMPs, and (ii) direct adjuvant delivery to APCs.

Photo-activated hybrids are a clean example. Exosome-encapsulated black phosphorus nanoparticles act as a photothermal/photodynamic core that triggers robust ICD, while the exosomal membrane improves intratumoral delivery and antigen capture, collectively boosting DC maturation and downstream CD8^+^ priming in multiple models (79). A complementary design uses tumor-exocytosed exosome/AIE luminogen hybrid nanovesicles that achieve deep tumor penetration before light activation; this sequence (penetration → ROS-mediated tumor cell death) markedly enhances photodynamic efficacy and is expected to increase the availability of tumor antigens *in situ* (80).

Cold-to-hot conversion can also be achieved by engineering the adjuvant directly onto/into exosome hybrids. In a representative glioma system, immune exosomes loading self-assembled therapeutic nanomicelles traverse the blood–brain barrier; once inside the brain, the combined chemo- and immunotherapeutic effects of the payload enhance glioblastoma control and promote local immune activation within the brain tumor microenvironment (81). Beyond exosome platforms, ferroptosis-augmented sonodynamic nanoplatforms further demonstrate that carefully tuned ROS/ferroptotic death can amplify sonodynamic tumor killing and provide a ferroptosis-augmented platform that could, in principle, be combined with immunotherapy (82). Exosome-coated nanoparticles for glioblastoma exemplify how cloaking inorganic or drug-loaded cores with tissue-homing exosomal membranes can improve intratumoral accumulation and treatment specificity (83).

### 5.2 Sensitizing immune checkpoint blockade and reversing resistance

Even in “hotter” tumors, ICI can fail because local suppressive cues and circulating exosomal checkpoints blunt T cells. Exo-nanomaterials attack both. First, hybrids can locally remove PD-L1 pressure. Biomimetic exosomal vesicles loaded with siRNA against PD-L1 have been used to inhibit tumor secretion of PD-L1-rich exosomes, thereby reducing systemic “decoy” suppression of PD-1^+^ T cells and improving CD8^+^ T-cell–mediated antitumor responses in preclinical models (84). Second, hybrids can reprogram suppressive myeloid checkpoints that sit upstream of PD-1 failure. Engineered exosome-like nanovesicles that promote tumor ferroptosis have been shown to remodel the tumor microenvironment and drive macrophages toward a more inflammatory, antigen-presenting phenotype, thereby creating conditions that are more permissive for effective checkpoint blockade (85). Together, these data suggest that exo-nanomaterials can create “ICI-ready TIMEs” by allowing checkpoint antibodies to act on a remodeled, less suppressive target.

### 5.3 Tumor vaccines and personalized antigen delivery

Exo-nanomaterials are especially well matched to vaccines because they can co-deliver antigen and adjuvant in the same lymph-node-draining particle, preserving native conformations and trafficking routes.

A clear personalized example is DC-derived exosome neoantigen nanovaccination, where exosomes loaded with neoantigenic peptide nanocomplexes drive strong CD8^+^ expansion and potent antitumor activity. The vesicular membrane promotes lymph node uptake and efficient cross-presentation compared with soluble peptides, improving primary tumor control and reducing metastatic burden in preclinical models (86). Complementarily, engineered exosome-like nanovesicles that reprogram the tumor microenvironment and promote ferroptosis provide a semi-biological vaccine-like effect: they carry tumor antigens while simultaneously modulating stromal and myeloid compartments, so that cytotoxic T lymphocytes (CTLs) attack both malignant cells and supportive stromal elements.

### 5.4 Combination therapies

Most successful exo-nanomaterial regimens now behave like “built-in combination therapies.” The hybrid supplies a physical trigger and a biological traffic map, so that killing and immune priming are inseparable in practice.

Phototherapy-driven exo-hybrids described above are not just cytotoxic; they are antigen-making machines that pulse danger signals at the exact time immune cells arrive. Thermosensitive exosome–liposome hybrid nanoparticles that co-deliver docetaxel and GM-CSF provide a similar proof-of-concept: under mild hyperthermia, a single vesicular platform can combine locoregional chemotherapy with systemic chemoimmunotherapy and significantly improve control of metastatic peritoneal carcinoma over chemotherapy alone (87). Sonodynamic and ferroptosis-coupled hybrids extend this logic to deep lesions by producing ROS and immunogenic death under ultrasound, while simultaneously reshaping local redox and lipid peroxidation pathways. Mechanistically, these systems illustrate a shared rule: the tighter the stimulus–killing–APC loop, the stronger the systemic immunity.

### 5.5 Representative evidence across tumor types

Evidence for immunotherapy-active exo-nanomaterials is no longer confined to one niche tumor. In glioblastoma, neural stem-cell-derived exosomes delivering CpG-STAT3 antisense oligonucleotides show that re-arming local myeloid/APC populations and relieving STAT3-driven suppression can convert a poorly immunogenic glioma into an immunotherapy-responsive state (88). In hepatocellular carcinoma, exosome-like photo-immunotherapy nanomedicines demonstrate that liver-tropic vesicular properties can be paired with external light triggers to induce potent intrahepatic tumor control and to remodel the immune microenvironment toward enhanced antitumor responses (89).

In fibroblast-rich solid tumors, ferroptosis-promoting exosome-like nanovesicles highlight a distinct advantage of exosome-mimetic hybrids: by targeting both tumor cells and suppressive stromal/myeloid components, they open the stromal barrier and increase ICI accessibility. Finally, in melanoma, breast and colon models, tumor-exosome-bionic nanozymes based on Type-I AIE photosensitizers induce ICD-associated DAMP release, promote DC maturation and enhance CD8^+^ T-cell–mediated antitumor immunity (90).

## 6 Conclusions and discussion

Exo-nanomaterials sit at the intersection of two rapidly maturing fields: exosome biology and cancer nanomedicine. Recent overviews of hybrid nanomaterials and nano-immunotherapy emphasize that combining immune-modulatory cargo with programmable carriers can convert otherwise marginal responses into durable tumor control, particularly when delivery is tailored to the TIME rather than to the tumor bulk alone (91, 92). These data support the central premise of this review: exosomes provide a biologically privileged interface with immune and stromal compartments, while synthetic nanomaterials contribute scalable loading, spatiotemporal control and multifunctionality; their integration offers capabilities that neither modality can achieve independently.

On the vesicle side, exosome-based delivery has moved beyond conceptual proof into increasingly sophisticated “smart” designs. Exosome-inspired platforms now routinely integrate targeting ligands, controlled-release modules and imaging handles within a single vesicle, demonstrating improved drug penetration, reduced systemic toxicity and better orchestration of immune activation in preclinical models (93). Parallel work on how EVs remodel the TIME shows that EV-mediated crosstalk can either propagate immune suppression or distribute antitumor signals across spatially separated niches (94). Together, these findings underscore a key translational lesson: therapeutic exo-nanomaterials must be constructed with explicit consideration of endogenous EV signaling circuits, or they risk reinforcing the very suppressive programs they are meant to overcome.

From the nanomaterial perspective, the last few years have clarified both the promise and the liabilities of nanoparticle-driven immunotherapy. Systematic analyses of nanoparticle platforms highlight their ability to co-deliver antigens, adjuvants and checkpoint modulators, but also caution that particle composition, size and surface chemistry can trigger unintended innate sensing or off-target accumulation (95). At the same time, focused reviews of STING agonist delivery reveal that rational nanoformulation is often the difference between intolerable systemic cytokine storm and therapeutically useful, tumor-restricted interferon programs (96). For exo-nanomaterial design, these insights argue for “immune-by-design” engineering: the inorganic or polymeric core must be optimized not only for drug loading and release, but also for a predictable and acceptable pattern of innate immune engagement.

Locoregional depots provide an additional layer of control that is highly compatible with exo-nanomedicine. Hydrogel-based delivery systems have emerged as versatile platforms for sustaining local concentrations of immunomodulators, promoting lymphoid drainage of antigens and minimizing systemic exposure (97). When exosomes or exo-coated nanoparticles are embedded in such hydrogels, it becomes possible to stage immune activation in a temporally extended fashion: early waves of ICD and antigen release can be followed by prolonged adjuvant and checkpoint modulation within the same anatomical space. Conceptually, this suggests a future in which exo-nanomaterials function not only as mobile carriers but also as modular components of injectable or implantable immune “micro-reactors” in the surgical cavity or tumor bed.

Sources of vesicles also deserve broader consideration. Plant-derived extracellular vesicles (PDEVs) and other non-mammalian EVs are being explored as low-cost, scalable and potentially less immunogenic carriers for nucleic acids and small molecules (98). Their natural stability across gastrointestinal and systemic routes, together with the possibility of agricultural-scale production, offers an attractive route to resolving current manufacturing bottlenecks in exosome therapy. However, differences in lipid composition, glycosylation and innate sensing raise unresolved questions about long-term safety, biodistribution and interaction with human immune cells. For exo-nanomaterials, PDEVs and other “cross-kingdom” vesicles may ultimately serve as customizable shells that combine unique tissue tropism with synthetic cores, but this will require much tighter mapping of their immunological fingerprints.

A recurring theme across successful platforms is the importance of spatiotemporal precision in immune stimulation. “Smart” on-demand release systems that respond to pH, enzymes, redox potential or external physical triggers can align antigen exposure, innate sensing and effector recruitment in ways that fixed-dose regimens cannot (99). Sequential delivery systems push this logic further by decoupling early priming signals (e.g., ICD inducer plus antigen capture) from later maintenance signals (e.g., checkpoint modulation or cytokine support) within one integrated carrier (100). When such sequential or stimulus-responsive strategies are implemented in exo-nanomaterials, they offer a route to dynamically adapt to TIME evolution—for example, by front-loading myeloid reprogramming in “cold” lesions and postponing T-cell boosting until sufficient antigen presentation has been restored.

Overall, the available evidence supports cautious optimism. Exo-nanomaterials have demonstrated the capacity to (i) enhance antigen visibility, (ii) concentrate potent innate agonists at otherwise inaccessible sites, and (iii) rewire suppressive myeloid and stromal compartments, thereby sensitizing tumors to checkpoint blockade and other systemic therapies. At the same time, major challenges remain, including standardizing vesicle isolation and characterization, deconvolving the contributions of exosomal versus synthetic components in complex hybrids, ensuring batch-to-batch reproducibility at clinical scale, and defining long-term safety in immunologically diverse patient populations. In the near term, translationally viable exo-nanomaterials will likely favor compositionally defined cores and standardized membrane sources, with critical quality attributes (CQAs) that link membrane integrity, targeting ligands, and immune readouts to reproducible *in vivo* activity.

Future progress will likely depend on iterative feedback between high-resolution TIME profiling, quantitative modeling of vesicle immune interactions, and carefully controlled early-phase trials. If these components can be aligned, exo-nanomaterials are well positioned to move from experimental tools to a coherent class of next-generation immunotherapies capable of addressing the spatial, temporal, and ecological complexity of solid tumors.
